# Legal and ethical issues of using brain imaging to diagnose pain

**DOI:** 10.1097/PR9.0000000000000577

**Published:** 2016-11-30

**Authors:** Karen D. Davis

**Affiliations:** Department of Surgery and Institute of Medical Science, University of Toronto; Division of Brain, Imaging and Behaviour-Systems Neuroscience, Krembil Research Institute, Toronto Western Hospital, University Health Network, Toronto, Ontario, Canada

**Keywords:** Chronic pain, Brain imaging, fMRI, Neuroethics, Biomarker, Nociception, Dynamic pain connectome

## Abstract

Pain, by definition, is a subjective experience, and as such its presence has usually been based on a self-report. However, limitations of self-reports for pain diagnostics, particularly for legal and insurance purposes, has led some to consider a brain-imaging–based objective measure of pain. This review will provide an overview of (1) differences between pain and nociception, (2) intersubject variability in pain perception and the associated brain structures and functional circuits, and (3) capabilities and limitations of current brain-imaging technologies. I then discuss how these factors impact objective proxies of pain. Finally, the ethical, privacy, and legal implications of a brain-imaging–based objective measure of pain are considered as potential future technological developments necessary to create a so-called “painometer test.”

## 1. Introduction

Chronic pain has tremendous personal and societal cost, with hundreds of millions of sufferers and enormous costs resulting from treatment and lost wages.^24,31^ In 2010, the International Pain Summit of the International Association for the Study of Pain put forth the Declaration of Montreal,^25^ which declares the rights of people with pain, highlights the right to “access to pain management,” “acknowledgement of their pain,” and “access to appropriate assessment and treatment of the pain.” Furthermore, the Declaration stated that assurance of these rights required: “the obligation of governments and all health care institutions… establish laws, policies, and systems that will help to promote, and will certainly not inhibit, the access of people in pain to fully adequate pain management.”

However, the inherently subjective nature of pain can be a roadblock to confirm its existence; thus, creating a hardship for patients who must “prove” they have chronic pain. The subjective nature of pain also poses a challenge for health care providers, insurance companies, and legal actors who need information about a patient or claimant's pain state to provide appropriate pain management, health care, and financial support.

This clinical and societal need and advances in magnetic resonance imaging (MRI)–based brain imaging have brought to the fore the issue of whether there are brain biomarkers of pain that can be used to diagnose and verify the presence of chronic pain. Thus, a so-called “painometer” objective test of pain based on brain imaging is currently being sought after. However, the use of any medical test raises a myriad of associated ethical issues.

This review will discuss ethical, privacy, and legal implications of a brain-imaging–based objective measure of pain. I will then provide an overview of the factors that impact objective measures of pain including (1) differences between pain and nociception, (2) intersubject variability in pain perception and the associated brain structures and functional circuits that represent pain, and (3) capabilities and limitations of current brain-imaging technologies. Potential future technological developments that are necessary to create a so-called “painometer test” will also be noted.

This review derived from the Refresher Course on Neuroimaging of the International Association for the Study of Pain 16th World Congress on Pain in Yokohama (Japan) in 2016 and contains large portions of the refresher course chapter emanating from it.^8^

## 2. A “painometer” landmark legal precedent

We are all familiar with the use of fingerprinting as a test of someone's identity. This test has been used for over 100 years and is based on the presumption of a unique anatomical pattern of indentations on our fingers. Despite the century-old and widespread use of fingerprinting, a special committee of the National Academy of Science recently declared that fingerprints and other forensic evidence lack the degree of reliability that has long been assumed.^16,38^ There is also a long history of tests meant to assess a person's thoughts and feelings. For example, the polygraph measures a person's physiological responses to questions and is purported to be a lie detector. Modern approaches to lie detection have also been developed using electroencephalography and functional MRI (fMRI). However, neither the polygraph nor the brain-based tests are generally accepted by the courts, owing to a range of confounds that can lead to false positives and false negatives.

Despite this long legal battle against the use of objective measures of internal thoughts, a recent case in a U.S. court has found admissible an fMRI-based test to verify the presence of chronic pain. The case involved a man (Carl Koch) who sustained a workplace injury that resulted in severe first- and second-degree burns to his right arm. Mr. Koch sued his employer for damages associated with his claim of intractable chronic neuropathic pain. Mr. Koch underwent an fMRI test to assess differences in brain activation when stimuli were applied to his right (affected) vs left (unaffected) arm. Dr. Joy Hirsh, a cognitive neuroscientist at Yale University who conducted the test, claimed that a different pattern of brain responses was evoked by stimulation of his right vs left arm and submitted her findings as evidence in support of Mr. Koch's subjective claim of pain. The judge deemed the fMRI “painometer” test as admissible, and Mr. Koch's case was settled for significantly more money than his employer had originally offered. Troubling to the scientific community^6,32^ was that this “painometer test” was accepted although it (1) was conducted and analysed by a neuroscientist without experience in or understanding of the pain field; (2) examined evoked and not ongoing pain; (3) lacked any evidence to link Mr. Koch's chronic pain with any specific brain response; (4) lacked control conditions to rule out nonspecific activations (eg, due to salience); (5) lacked countermeasures for deception; and (6) did not provide any evidence that the data analysis met standards of statistical rigor, was repeatable, robust, and represented an abnormal response compared with healthy individuals. This landmark case has raised serious neuroethical issues that are discussed below.

## 3. The neuroethics and consequences of adopting a “painometer”

The use of a brain-imaging test to determine whether someone does or does not have pain is tantamount to a lie detection test and has associated neuroethical and privacy implications. Requiring such a test conveys the sentiment that patients may be dishonest in their claim to be experiencing pain. Thus, at its very core, a medicolegal system that calls for a so-called “painometer” test is placing the onus on patients to “prove” that their pain is real. Doubting a patient's self-report sets up a sense of distrust and can cause great stress to the patient and his or her friends, family, and coworkers. There are also societal and ethical issues associated with requiring a test to validate a patient's claim of pain. For example, brain imaging is costly and is not normally available outside major cities. Furthermore, many people cannot undergo fMRI (eg, if they have a pacemaker or other internal ferromagnetic devices, are pregnant, severely claustrophobic, or have vascular reactivity deficits). Thus, financial and accessibility issues preclude the universal use of a “painometer” test. Privacy of data pertaining to brain structure and function is another issue that must be carefully examined if brain imaging is to be acquired for the purposes of pain diagnosis. The issues are not unlike those associated with the collection of genetic information because this type of information is a window into risk factors and current and future personal behavior and functioning. Some countries have laws that protect personal privacy and safeguard against the misuse of genetic information, but equivalent protection pertaining to brain data is not yet well-developed.

**Figure 1.**
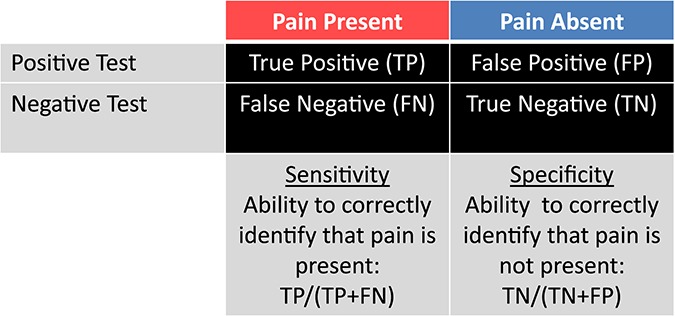
The ability of a brain-based objective measure of pain to correctly identify whether or not pain is present in an individual is based on the sensitivity and specificity of the test.

All medical tests have a certain degree of sensitivity and specificity based on true-positive and true-negative rates (Fig. 1). There are certainly potential benefits to developing an objective measure of and proxy for pain with very high sensitivity and specificity.^14^ For example, such a test would serve to validate patients' self-report so that they can proceed through the medicolegal system to receive the care that they need. Furthermore, in theory, the specific findings of a particular pattern of brain abnormalities could optimize the choice of a treatment plan for each patient with the best chance of success and minimal side effects, thus, leading to personalized and precision pain management. The test would also be enormously beneficial to identify pain in situations where the patient cannot provide a self-report.

Nonetheless, false-negative and false-positive outcomes of a “painometer” test can have serious consequences and waste resources.^14^ A false-positive result (ie, concluding that someone has pain when he or she does not) may seem to be relatively harmless, but it does have deleterious effects. Arguably, the greatest issue pertaining to false-positive findings is the unwarranted settlements that would be paid to the claimant. In insurance claims and medicolegal cases, these costs have a negative impact on employers and could drive up insurance rates and health care costs. Further, if a “painometer” test is being used to direct pain management toward a particular brain abnormality, an erroneous positive finding could initiate potentially harmful and inappropriate treatments.

However, a false-negative finding is the greatest concern of “painometer” tests because of the potential medical, family, workplace, and financial consequences. For example, if a patient is truly suffering from a chronic pain condition, a test that refutes that truth could be used to deny the patient proper treatment, contravening the Declaration of Montreal.^25^ Beyond medical consequences, a false-negative finding also adds undue stress for patients, and produces trust issues that can affect their relationships with family, friends, colleagues, their health care team, and their employer. These issues in turn can affect mental health and negatively affect employment and insurance conditions—all contributors to financial hardships.

## 4. Pain is subjective: can nociception be a proxy?

Pain, by definition, is a subjective experience,^23^ and as such it is typically determined by self-report.^11^ The medical–legal community can be suspicious of pain self-reports in cases of “idiopathic” chronic pains in which there is no clear understanding of pathology. The issue of honesty also becomes a factor in situations where there is financial gain, such as in personal injury claims. Furthermore, there are many instances in which a self-report cannot be obtained (eg, in noncommunicative individuals or circumstances), and so there is utility in proxies of pain.

Compared with pain, there is a related but not-equivalent concept of nociception, which is defined by the International Association for the Study of Pain as “the neural process of encoding noxious stimuli.” Nociception is the overall nociceptive activity in the brain, which is an activity evoked by noxious stimuli. This activity has been used as a proxy for pain. The concept of brain-based objective measure of pain relies on the ability to measure nociceptive activity and its suitability to substitute for pain self-reports. Clearly, pain and nociception are not equivalent, as is evident from the fact that they can be disconnected. For example, pain is not experienced under anesthesia, even though nociceptive activity can still be recorded in the brain. Also, painful stimuli elicit brain activity related not only to pain percepts but also to cognitive and motor functions for evaluation and response to the stimulus. Furthermore, equating pain with any measure of nociception that we currently have available is fraught with problems owing to the complexity of the pain experience and the sharing of all brain real estate that encodes pain with nonpain functions.^22^

## 5. Major obstacles to developing a brain-imaging–based test of pain

There are enormous challenges in establishing and being able to detect a “chronic pain biomarker” with a high degree of certainty in an individual person. Some of the key issues are described below.

### 5.1. Representation of pain in the brain

**Figure 2.**
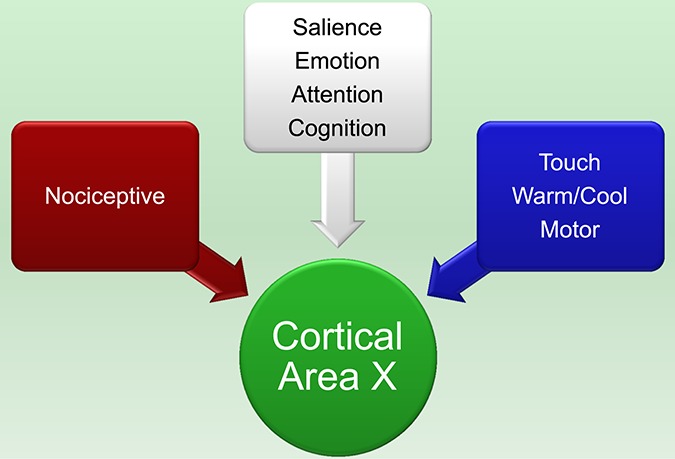
Areas of the brain that receive nociceptive input also receiving input from nonnociceptive systems.

It is well established that pain does not reside in any 1 particular part of the brain but rather engages a distributed system.^1^ Furthermore, every area of the brain that responds to noxious stimuli or is linked to pain perception is also involved in other functions such as touch, attention, salience, emotion, and other cognitive and sensorimotor functions^22^ (Fig. 2). The recent concept of a dynamic pain connectome^27^ highlights the fact that pain engages not just the areas traditionally associated with ascending nociceptive pathways (eg, somatosensory, insular, cingulate, and prefrontal cortices) but also brain networks underlying attention and salience and the default mode network. Although the activity in some brain areas shows associations with particular aspects of pain (notably pain intensity), this does not necessarily mean that these areas are specific to pain. This issue is at the core of the concept of a “pain switch” that acts as an ouch detector.^13^ This distributed and nonspecific representation of pain poses an enormous roadblock to establishing a valid “painometer.” Emerging computational work is promising to solve this problem by decoding brain imaging data so that the presence of pain in an individual can be predicted on the basis of multivoxel pattern analysis and machine learning algorithms.^2,4,20,35,39^ Thus far, most of these studies have only examined acute pain responses in small groups of young healthy adults. Although a small number of studies have examined chronic pain, these studies either evaluated fMRI of evoked pains or used structural MRI and did not have adequate sensitivity or specificity.^3,30,33^ Thus, it is not known whether such approaches can be developed to detect ongoing chronic pain rather than evoked responses and be valid across a wide spectrum of individuals with a high degree of sensitivity and specificity.

### 5.2. Intersubject variability in pain perception and brain structure and function

It is well known that among healthy men and women, there is vast intersubject variability across all aspects of the pain experience, including threshold and suprathreshold measures of pain, qualities of pain evoked by acute stimuli, and pain-modulatory responses (eg, Refs. 5,^7^,^19^,^26^,^42^). There is also considerable intersubject variability in how much pain interferes with the ability to perform cognitive tasks. We have characterized this variability into 2 categories: P-type individuals (pain dominates), who perform a task slower when there is concurrent pain, and A-type individuals (attention dominates), who give a higher priority to the task and, thus, show faster performance during concurrent pain.^17,29,37^ We also have discovered that the ability of an acute pain stimulus to capture one's attention varies widely, with some people mind-wandering from the pain and others engaged with it.^29^ The intersubject variability in pain threshold, suprathreshold metrics, and these attentional responses have been linked to variability in brain structure and function.^17,18,29,34,37,40^ In addition, it is well known that situational conditions (eg, stress) and individual traits and psychological factors contribute to sensitivity and brain structure and function (eg, Refs. 28,^36^). Thus, it is clear that a “one size fits all” biomarker of chronic pain cannot be simply constructed. Rather, a valid model of chronic pain can only be constructed when an appropriate model of acute pain is derived that considers vast intersubject variability among the healthy population.

### 5.3. Magnetic resonance imaging technical and statistical issues

The reliability and repeatability of any sort of “painometer” test requires standardization of the type of equipment used, the way in which the test is performed, and the analysis of the data (Table 1). For example, the final result of a test is enormously dependent on a variety of factors that affect the data signal-to-noise characteristics, together with the resultant spatial and temporal resolution of the data. Thus, a standardized test must consider the choice of the MRI machine and its field strength, the pulse sequence and acquisition parameters used to acquire the data, the type of preprocessing, models, and statistical analyses. In fact, one of the most contentious issues in fMRI is the choice of what level of statistical significance should be used.

**Table 1:**
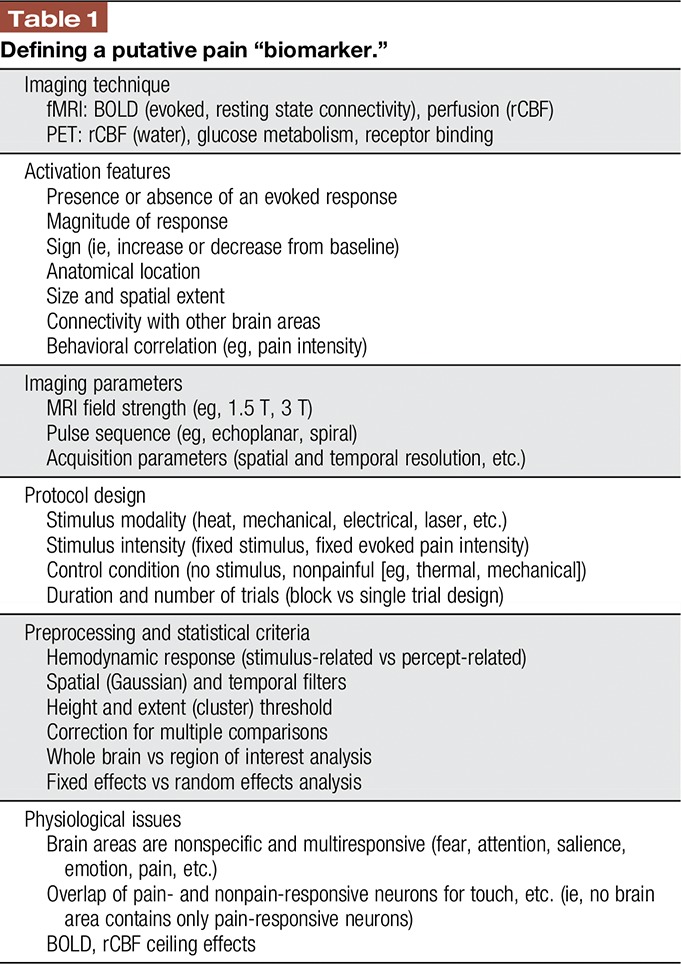
Defining a putative pain “biomarker.”

### 5.4. Physiological issues

It is important to keep in mind that fMRI signals are not direct measures of neuronal activity but rather arise from the slow hemodynamic functions associated with neuronal activity on the order of several seconds. Thus, the actual frequencies of neuronal firing that occur on a millisecond timescale are not detectable with fMRI. Functional magnetic resonance imaging provides a much higher-level global view of brain activity and connectivity. Furthermore, changes in blood flow and deoxyhemoglobin/oxyhemoglobin are not infinite, and so there is a ceiling effect that can preclude the detection of neuronal activity in certain circumstances. For example, if an area of the brain contains some neurons that are very active, additional activity in those neurons or nearby neurons may not be detectable (see Ref. 9). This issue is an important one yet is rarely discussed in the field of pain. However, it has serious consequences because all brain areas that contain nociceptive neurons also contain nonpain neurons, and so without careful control conditions, it may not be possible to detect a pain-specific response. For example, the human mid-cingulate cortex (also referred to as the dorsal anterior cingulate cortex) contains both nociceptive neurons^21^ and neurons activated by attention-demanding^12^ and emotional stimuli.^15^ Even with careful controls, it still may not be possible to disambiguate pain-specific from nonspecific responses because (1) additional metabolic needs may not translate into detectable additional blood-oxygen-level–dependent signals and (2) most situations of pain also engage salience, attention, emotion, and other systems. Furthermore, as a consequence of ceiling effects, in situations of chronic pain when nociceptive neurons are presumably active, the additional activity evoked in those neurons during a hyperalgesic protocol may not be detectable. Another rarely considered issue is that the blood-oxygen-level–dependent response is tied to a healthy vascular system, and there are situations in which vascular reactivity may be compromised (eg, in poststroke patients or the elderly).

### 5.5. Defining and testing a brain biomarker for chronic pain

There is currently no brain-imaging–based biomarker for chronic pain. There are several challenges to establishing a chronic pain biomarker (Table 2). Toward this goal, it is imperative to have knowledge of the full range of brain features related to pain in healthy individuals, as noted above. This “range of normal” must also include different ages, sex/gender, ethnic/racial background, and other individual factors. However, to date there are only small-scale studies of this nature.

**Table 2:**
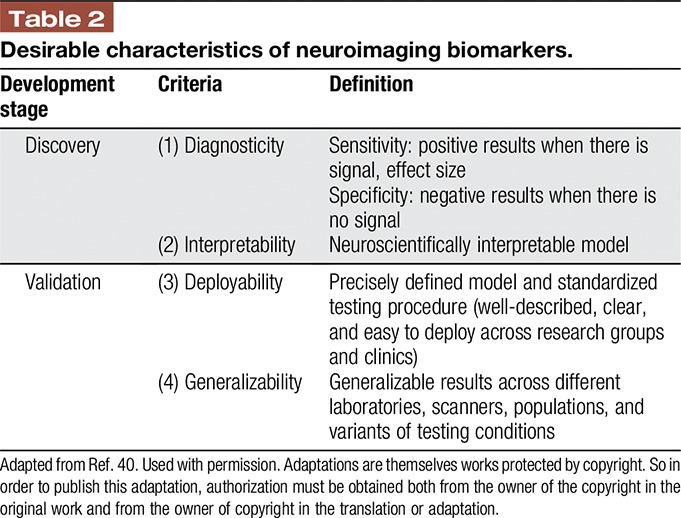
Desirable characteristics of neuroimaging biomarkers.

In the case of allodynia or hyperalgesia, one must be able to determine whether a person shows an abnormal response to acute stimuli. Responses may vary for different types of stimuli (heat, cold, mechanical, or chemical), different body sites, and under different experimental conditions. Furthermore, an fMRI response and differences between subjects can be characterized by many features, such as anatomical location, signal magnitude and sign (activation and deactivation), spatial extent, and correlation against a behavioral or stimulus attribute (see Ref. 10), none of which have been considered to be the gold standard in defining a normal or abnormal condition.

The most pressing issue in chronic pain detection is that chronic pain is characteristically present without an overt stimulus. Thus, there is a need for imaging approaches other than the typical stimulus-evoked fMRI paradigms. Potential approaches include resting-state functional connectivity or perfusion methods such as arterial spin labeling or positron emission tomography. Despite some experience with these technologies, they have not yet been developed to the degree that they can identify a chronic pain state.

If a neuroimaging biomarker is developed, it must be rigorously tested to meet a high standard of specificity, sensitivity, and validity. The basic criteria suggested for this purpose include diagnosticity, interpretability, deployability, and generalizability^41^ (Table 2). It is unlikely that a brain-based biomarker of pain will supplant or totally replace the current gold standard of self-report in all situations. Rather, such a biomarker could serve as an adjunct to self-report to better understand the brain circuits that are associated with pain. In this context, an objective brain measure that serves as a proxy of pain would be useful as a research tool and possibly to guide clinical management of pain.

## 6. Conclusions

The last few years have seen a tremendous effort toward developing a brain-imaging–based model of pain. This academic pursuit can lead to new knowledge about how pain is represented in the brain in health and disease. We are also learning about individual nuances that contribute to pain sensitivity and vulnerabilities which provide insight into the development and intractability of chronic pain. New sophisticated approaches (eg, machine learning) hold the promise of modeling the complexities of general acute pain representations in the brain. However, our current understanding of the brain signatures representing chronic pains across individuals is in its infancy. Thus, shifting these academic research endeavors into a real-world practical and clinical setting (in particular for diagnostics used in the legal setting) is premature at this time. However, if a “painometer” becomes technically and scientifically possible, it is imperative to carefully consider and take safeguards against the far-reaching ethical and legal implications of such a test.

## Conflict of interest statement

The author has no conflicts of interest to declare.
